# Green Synthesis of Metallic Nanoparticles Using Some Selected Medicinal Plants from Southern Africa and Their Biological Applications

**DOI:** 10.3390/plants10091929

**Published:** 2021-09-16

**Authors:** Jumoke A. Aboyewa, Nicole R. S. Sibuyi, Mervin Meyer, Oluwafemi O. Oguntibeju

**Affiliations:** 1 Oxidative Stress Research Centre, Phytomedicine and Phytochemistry Group, Department of Biomedical Sciences, Cape Peninsula University of Technology, Bellville 7535, South Africa; Jumokeaboxe@gmail.com; 2 Department of Science and Innovation (DSI)/Mintek Nanotechnology Innovation Centre, Biolabels Node, Department of Biotechnology, University of the Western Cape, Bellville 7530, South Africa; nsibuyi@uwc.ac.za

**Keywords:** green nanotechnology, metallic nanoparticles, medicinal plants, antimicrobial, cytotoxicity, green synthesis

## Abstract

The application of metallic nanoparticles (MNPs), especially that of silver, gold, cobalt, and zinc as antimicrobial, anticancer, drug delivery, contrast, and bioimaging agents has transformed the field of medicine. Their functions, which are attributed to their physicochemical properties, have gained prominence in various technological fields. Although MNPs can be produced via rigorous physical and chemical techniques, in recent years, a biological approach utilizing natural materials has been developed. With the increasing enthusiasm for safe and efficient nanomaterials, the biological method incorporating microorganisms and plants is preferred over physical and chemical methods of nanoparticle synthesis. Of these bio-entities, plants have received great attention owing to their capability to reduce and stabilize MNPs in a single one-pot protocol. South Africa is home to ~10% of the world’s plant species, making it a major contributor to the world’s ecological scenery. Despite the documented contribution of South African plants, particularly in herbal medicine, very few of these plants have been explored for the synthesis of the noble MNPs. This paper provides a review of some important South African medicinal plants that have been utilized for the synthesis of MNPs. The enhanced biological properties of the biogenic MNPs attest to their relevance in medicine. In this endeavour, more of the African plant biodiversity must be explored for the synthesis of MNPs and be validated for their potential to be translated into future nanomedicine.

## 1. Introduction

Over the years, MNPs (at a size range between 1 and 100 nm) have fascinated scientists and are currently utilized in various fields not limited to medicine, agriculture, and engineering [1,2,3]. The widespread practical applications of nanomaterials are attributable to their unique optical, catalytic, electronic, and physical properties [4,5]. The synthesis of MNPs with desired characteristics can be achieved via a variety of physical and chemical processes [6,7,8]. However, these methods are elaborate, expensive, time-consuming, and potentially hazardous to the environment and living organisms. The urgency to minimize the potential negative impacts of the nanomaterials synthesized via physical and chemical routes has led to the exploration of biological entities. The capability of microorganisms and plants to transform metal ions into MNPs revealed a simple, rapid, cost-effective, and eco-friendly approach for nanoparticle synthesis [9,10,11]. Microorganisms, including bacteria, fungi, and yeasts, can reduce metallic salts into nanoparticles (NPs) [12,13,14]. These microbes produce proteins, enzymes, reducing cofactors, peptides, and organic materials that play significant roles in the reduction of metallic salts into MNPs. These molecules can serve as either reducing and/or capping agents. In this regard, numerous microorganisms including *Klebsiella pneumoniae*, *Escherichia coli*, *Pseudomonas aeruginosa*, and *Candida albicans* have been used for the synthesis of MNPs [13,15,16]. Plants are by far the most important biological components for MNP synthesis, as their universal abundance and lack of pathogenicity offer an advantage over other biological sources. Plant-mediated synthesis is relatively mild, eco-friendly, cost and time effective; with their phytochemicals and bioactive contents acting as reducing, capping, and stabilizing agents [17,18]. Consequently, natural resources are conserved, and, at the same time, opportunities are created for sustainable development. Numerous studies have reported the successful synthesis of biogenic NPs using different plant species [6,8,19]. At their nanoscale size, the NPs exhibit significantly different characteristics, as well as improved bioactivity, compared to their bulk counterpart [6,20]. Despite the increasing awareness and promising benefits reported for plant-mediated NPs, this area of research remains underexplored. Of a fact, more than 80% of the world’s population rely on herbs/medicinal plant for treating diverse kinds of diseases, including diabetes, high blood pressure, cancer, and tuberculosis [21]. Moreover, nearly 60% of all synthetic drugs used for clinical purposes are derived from plants which further justifies the significance of medicinal plants [22].

South Africa houses about 10% of the world’s plant species, with over 3000 plants reported to have significant medicinal benefits [23]. Despite this biodiversity and the promising benefits of plant-mediated MNPs, the utilization of South African medicinal plants for the synthesis of MNPs is still largely underexplored [24]. Recently, indigenous South African plants including *Salvia africana-lutea* [6], *Sutherlandia frutescens* [6], *Galenia africana* [25], *Catharanthus roseus* [26], *Hypoxis hemerocallidea* [27], *Cotyledon orbiculata* [28], and *Aspalathus linearis* [29] were utilized for the fabrication of MNPs. These MNPs with sizes ranging from 5–50 nm were reported to exhibit high antibacterial activities compared to their respective plant extracts. Therefore, considering the vast potential of plants as alternative sources of reducing agents with enhanced bioactivities, continuous research towards the exploration of South African medicinal plant reserves will be of paramount importance.

The present review provides a brief overview of some potent South African medicinal plants utilized for nanoparticle synthesis. Characterizations, as well as the biological applications of the biogenic MNPs, were highlighted. With this update, rigorous research could be directed towards African medicinal plants to produce natural and effective nanoproducts that will revolutionize many technologies and industries, including pharmaceuticals, food, cosmetics, construction, medicine, engineering, and many others.

## 2. MNPs and Their Application

Nanotechnology is an important aspect of science that relies on the synthesis, modification, modulation, and application of materials within the nanometer scale (1–100 nm) [30,31,32]. NPs have attracted much interest in the last decades due to their distinct physicochemical, optical, magnetic, and biological properties [30]. Their properties, predominantly convened by their large surface area to volume ratio and size, are absent in bulk materials. Consequently, these unique properties are explored for application in environmental, water, food, biomedical, and space industries [4,33]. MNPs particularly those of noble metals such as silver and gold NPs have been widely explored in various biomedical fields [34] not limited to tissue engineering, health care, drug delivery, and gene delivery [34,35,36]. This section will give a brief overview of some of the most widely used MNPs and their application.

### 2.1. Silver NPs (AgNPs)

AgNPs have drawn considerable research interest due to their superior physical, chemical, and biological characteristics [26,37,38]. Rigorous efforts have been made to explore their integral properties for practical and clinical applications, particularly, as therapeutic and diagnostic agents [27,28]. Previous and recent discoveries have shown that AgNPs exhibit interesting antimicrobial effects against a wide range of microorganisms [6,28]. AgNPs are thus used in water purification and wound dressing as antimicrobial agents [35]. Their use for the production of paints, disinfectants, and some kitchen appliances has also been reported [37,39]. The therapeutic applications of AgNPs in terms of their antiviral, antifungal, anticancer, and antibacterial properties have also been demonstrated [6,27,28]. Currently, AgNPs are commercialized as antimicrobial agents in the pharmaceutical and cosmetic industries and are also utilized to protect against infections in various medical implants or bone cement [40,41].

### 2.2. Gold NPs (AuNPs)

AuNPs, on the other, hand have attracted significant interest over the last decades owing to their optical and chemical properties. Their potential as diagnostic and therapeutic agents in a variety of medical fields has been documented [42,43,44]. AuNPs are used as drug carrier/delivery, bioimaging, contrast, photothermal, and anti-angiogenic agents [45,46]. AuNPs were investigated for antibacterial activity and found to display an effective antibacterial effect against gram-positive and gram-negative bacterial strains [47,48,49,50]. AuNPs can ferry and deliver hydrophobic and hydrophilic drugs, peptides, antibodies, and small molecule drugs to the targeted tumour site with no toxic effects on normal or surrounding tissues [51]. Moreover, AuNPs provide a platform to attach multiple moieties on their surface, therefore, they are suitable as drug delivery agents [4,52]. AuNPs can be functionalized with therapeutic moieties to exhibit enhanced anticancer effects [51,53]. Additionally, strategies that involve attaching targeted peptides that would possibly target receptors that are exclusively expressed by diseased cells have been reported [53]. AuNPs-conjugates were reported to significantly enhanced the selectivity as well as the sensitivity of therapeutic peptides on pancreatic (Panc-1) and colon (Caco-2) cancer cells [53,54]. According to previous and recent studies, biosynthesized AuNPs have achieved significant targeting and selectivity against a variety of cancer cells without any additional molecules [19]. A study by Majoumouo et al. [19] demonstrated that the AuNPs synthesized using *Terminalia mantaly* exhibited enhanced cytotoxicity and selectivity on Caco-2, epithelial breast (MCF-7), and liver (HepG2) cancer cells. Another study by Anadozie and co-workers [19] revealed that AuNPs synthesized from water extract of *Xylopia aethiopica* showed excellent anticancer activity on MCF-7 and Caco-2 cells. These reports and many more have opened a limitless opportunity towards the incorporation of AuNPs in molecular diagnostics and therapy.

### 2.3. Metal Oxide NPs

Copper and copper (II) oxide NPs have found diverse applications in physics and material science engineering. They are strong antimicrobial agents exhibiting excellent disinfecting properties against several infectious organisms [55]. The application of iron oxide NPs in many biomedical applications like gene therapy, stem cells, cancer, and atherosclerosis has been reported [56]. These NPs have been developed as anticancer, antifungal, antimicrobial, and targeted drug delivery agents. Zinc oxide (ZnO) NPs are popularly known for their antimicrobial and anticancer activities. They have been found useful in food packaging, wastewater treatments, and some personal care products [13]. ZnO NPs were used as preservatives in food packaging to prevent and inhibit the growth of microbes on food materials. Moreover, ZnO NPs proffer toxicity against microorganisms compared to all other metal oxides NPs.

## 3. Synthesis of MNPs

**Figure 1:**
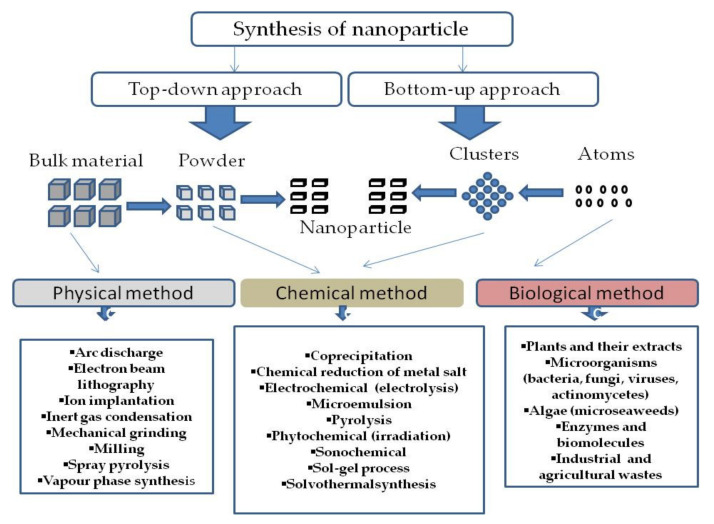
Methods used for the synthesis of MNPs. The different methods are classified as top-down and bottom-up. Adapted from [4].

Consequent to the continuous utility of MNPs in several modern-day applications, the method for their preparation needs to be safe to achieve better control of desirable physicochemical and bio-functional properties. Several methods and modifications have been explored for their synthesis [14]. Over the years, NPs have continuously been used and modified to enable their application in various fields ranging from agriculture and biomedicine. The techniques developed for the synthesis of NPs are classified as top-down and bottom-up. The top-down approach reduces bulk material of interest to NPs, while the bottom-up approach build-up smaller material into the required nanostructure [32]. Further, these techniques are classified into physical, chemical, and biological methods of NP synthesis as depicted in Figure 1. Physical methods mainly operate on a top-down strategy where bulk materials are systematically broken down bit by bit to produce finely divided NPs [4]. The physical methods rely on mechanical pressure, electrical and radiation energy, melting, evaporation, or condensation techniques to produce NPs. Examples include vapor condensation, aerosol, laser ablation, pyrolytic, high-energy ball milling, laser pyrolysis, inert gas condensation, and mechanical crushing processes [4]. The chemical methods involve the use of organic and inorganic substances, including sodium citrate, elemental hydrogen, sodium borohydride, hydrazine, dimethylformamide, and ascorbate as reducing agents for the synthesis of the NPs. Sol-gel, hydrothermal, chemical vapour deposition, microemulsion, and polyol techniques are the commonly used chemical methods [4].

Although the physical and chemical approaches are the most popular, they are costly, time-consuming, and require sophisticated working conditions. Moreover, their application, particularly in biomedicine, is threatened with toxicological effects on humans, animals, and the environment [57,58]. The search for non-toxic and environmentally friendly protocols which do not use hazardous chemicals and complicated physical techniques in NP synthesis has led to the exploration of biological entities. Thus, microorganisms and plants have emerged as green agents, providing eco-friendly routes for the synthesis of NPs.

### 3.1. The Advent of Green Nanotechnology

Green nanotechnology is an exciting and emerging area of science and technology that embraces the principles of green chemistry with potential benefits towards sustainability, protection, and overall safety of the human race [59]. The green chemistry methodology introduces a desirable approach to the synthesis, processing, and application of less hazardous chemicals to reduce threats to human health and the environment [60]. The approach requires an in-depth understanding of the raw materials, especially in terms of their fabrication into nanomaterials and the resulting bioactivities that pose little or no hazardous effects on humans and the environment. On this note, nanomaterials can be engineered from natural sources at our disposal with the assurance of minimized potential risks. The use of biological entities as reducing, capping, and stabilizing agents in the synthesis of MNPs is constantly gaining wide attention [25]. The method utilizes microorganisms, and plant products as reducing agents [11], presenting benign and eco-friendly conditions that can improve safety in humans and animals [31].

The exploration of microorganisms such as bacteria, fungi, algae, and viruses as reducing agents for MNP synthesis is becoming more popular among natural product researchers. Microorganisms reduce metallic ions into their corresponding NPs with the help of enzymes produced by their cellular metabolism in two ways. (1) Intracellular pathway, where the metal ions are trapped and reduced inside the microbes (2) extracellular pathway, where the ions are reduced on the microbial cell surface or in the medium [5,12]. Fungi, which are sometimes referred to as “bio-nano factories,” have reportedly been used to reduce gold ions to AuNPs because of their capability to secrete large amounts of enzymes and their ability to withstand metal toxicity to a more considerable extent [13].

Yeast, such as C.albicans and algae such as Sargassum wighti have been successfully used to synthesize stable AuNPs making them good candidates for NP production [30]. Bacteria are also potential bio-factories for NP production, as they can withstand stress exerted by heavy metal toxicity. Some bacterial cultures such as *Pseudomonas stutzeri*, *P. aeruginosa*, *Thiobacillus ferrooxidants*, and *E. coli* were used to synthesize monodispersed MNPs with excellent biomedical applications [2,61]. In another study, the culture supernatants of *K. pneumonia*, *E. coli*, and Enterobacte cloacae were used as reducing agents for the rapid biosynthesis of AgNPs [47,61]. Microorganisms have undeniably provided MNPs with significant antimicrobial and anticancer properties [12]. However, the elaborate experimental procedures in terms of isolation, culture preparation, and maintenance have strained this approach. This limitation and others have upsurged the synthesis of MNPs using plant sources. Thus, plant-mediated synthesis is becoming most prominent, offering a quicker and more manageable approach than microbial and other green sources [2,10,34].

The potential of plants to bioaccumulate heavy metals suggests their use in the transformation of metal ions into MNPs. Alfalfa sprouts were the first plant reportedly used for the synthesis of AgNPs [62]. Subsequently, different plant species along with a variety of bioactive compounds have been explored for the production of MNPs including gold, silver, zinc, iron, copper, and platinum. Studies have shown that the reduction and stabilization of MNPs are achieved through the action of diverse compounds like proteins, amino acids, polysaccharides, and phytochemicals like flavonoids, alkaloids, tannin, and polyphenols present in the plants [63,64]. With the plant-derived approach, synthesis and purification are quicker and easier compared to the microbial-mediated approach [2,62].

### 3.2. The Synthesis of Plant-Mediated MNPs

**Figure 2:**
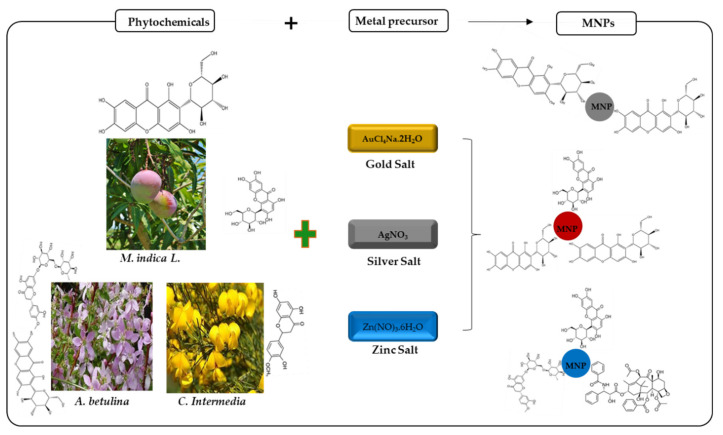
Synthesis of MNPs using plant extracts. Several plant extracts with high reductive capacity actively reduce metallic ions including silver, zinc and gold ions to their corresponding MNPs. Adapted from [20].

The notion that plants can bio-accumulate and reduce metal ions has opened options for considering their use as an alternative route for the synthesis of MNPs. The plant-mediated synthesis of NPs offers advantages over the microbes-mediated approach in simplicity, cost-effectiveness, rapidity, and non-pathogenicity [64]. Several plants including *Zingiber officinale*, *Punica granatum*, *Acalypha indica*, *Ficus benghalensis*, *Galenia africana*, *Terminalia mantaly*, and *Catharanthus roseus* have been reportedly used for the synthesis of MNPs. The synthetic process is initiated by the addition of extracts obtained from plant parts such as leaves, flowers, roots, stems, bark, and fruits into the aqueous solution of metal ions [8]. The phytochemicals present in the plant extracts which include sugar, flavonoids, protein, enzyme, polymer, and organic acid, act as reducing and stabilizing agents. The alkaloids, polyphenols, terpenoids, polysaccharides, amino acids, organic acids, vitamins, and heterocyclic compounds are implicated in the bioreduction process [64], and equally, play significant roles in the capping and stabilization of the bio-synthesized NPs. The exact mechanism and the plant components responsible for plant-mediated synthetic NPs remains complicated [8,19,20]. Several potential mechanisms have been proposed [65] as shown in Figure 2. During NP synthesis, a bioreduction phase occurs where bioactive compounds present in the plant extracts reduce metal ions/salts from their mono or divalent oxidation states to zero-valent states [66]. Subsequently, the reduced metal atoms’ nucleation indicated by physical observation of a colour change in the reaction medium takes place. As bioreduction and nucleation continue, a growth phase is reached where smaller particles mechanically interact to form larger particles that are thermodynamically more stable. The final stage of the synthesis is the termination stage, where the bioactive compounds exert their stability potentials and finally define the shape and morphology of the NPs.

Reports suggest that the mechanisms employed in the process of preparing NPs from medicinal plants differ from plant to plant due to the variation in their bioactive molecules, their composition and concentration [6]. The variation between plants and their subsequent interaction with aqueous metal ions as well as factors such as pH, temperature, and reaction time results in NPs exhibiting different physical, chemical, and biological properties [8]. Moreover, the properties of the phytochemicals present in the plants play the most vital role in the bioactivities of the NPs [28]. It is believed that the NPs derived from a particular plant extract may take on the bioactivities displayed by the plant extract [20]. In most cases, the NPs have a superior or higher bioactivity than the plant extract [19]. Such NPs can be engineered as nanoplatforms for molecular diagnosis, effective and targeted delivery of drugs, providing treatments of diseases that can resolve some of the drawbacks associated with traditional medicine [20]. These NPs have the potential to be translated into conventional medicine much faster, hence overcoming the many challenges associated with present-day standard modes of treatment.

## 4. Biological Application of MNPs Synthesized from Some Selected South African Medicinal Plants

### 4.1. South African Medicinal Plant Biodiversity

Africa is a continent endowed with remarkable natural biodiversity with over 45,000 different plant species documented [21]. In a comprehensive list containing African medicinal plants, only about 5400 plants have been reported to have medicinal properties. This represents approximately 12% of the total African plants and therefore supports the claim that African plants are under-utilized for medicinal purposes. The interest in Africa’s underexplored medicinal plants has recently upsurged; over 60% of recent publications focused on the African medicinal plants and their bioactivities [21]. Despite the increasing research interest, the commercialization of African plants still lags. A global review of commercialized medicinal plants revealed that only 83 African medicinal plants are considered partially or fully commercialized [23], whereas, in Europe and Asia, commercialization is at its maximum peak. Plants have been a major source of medicines in most African countries for thousands of years [67,68] and have played an integral role in basic traditional medicine and healthcare in many developing countries, including South Africa.

**Figure 3:**
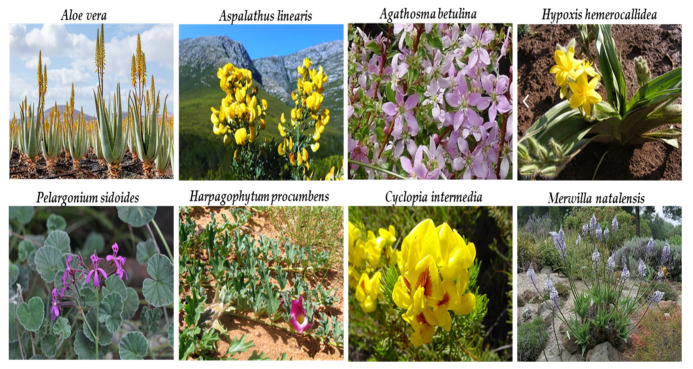
Catalogue of some of the indigenous plants found in the Cape Floristic Region of South Africa.

South Africa is the third most biodiverse country, and houses one of the world’s six floral kingdoms (the Fynbos Biomes) in addition to other biodiversity hotspots, the Succulent Karoo and the Albany-Maputaland corridor [23,69]. Figure 3 shows the floristic region of South Africa. According to the “African Plant Checklist and Database Project”, Sub-Saharan Africa houses 50,136 angiosperm taxa, out of which Southern Africa accounts for 22,755 taxa [23]. Moreover, South Africa hosts around 30,000 flowering plant species with over 3000 plants reported to have medicinal uses [21,68]. The global review of commercialized medicinal plants shows that South Africa accounts for 14 out of the 83 commercialized African medicinal plants [70]. The most valuable of all indigenous South African plants is the *Aloe vera* L. while those that have received universal recognition include *Agathosma betulina*, *Aloe ferox*, *Aspalathus linearis*, *Harpagophytum procumbens*, *Hypoxis hemerocallidea*, *Merwilla natalensis*, *pelargonium sidoides*, *Sclerocarya birrea*, and *Cyclopia intermedia*.

### 4.2. MNPs Synthesized from Indigenous SA Plants and Their Application

Medicinal plants have a long history in traditional medicine; over 80% of the South African population relies on the plants to meet their primary health care needs [68]. Medicinal plants offer differential therapeutic properties based on their integral supply of secondary metabolites, viz, flavonoids, alkaloids, phenolics, terpenoids, tannins, glycosides, quinones, steroids, and saponins [71]. However, most of these compounds have low absorption, resulting in a loss of bioavailability and efficacy due to their inability to cross the lipid bilayer of cells [72]. Thus, several phytochemical and pharmacological investigations of these plants and their derivatives revealed impressive in vitro activity and less in vivo efficacy. To facilitate effectiveness, one approach that is increasingly suggested in the literature is the combination of traditional medicinal plants with nanotechnology [73,74]. Nanotechnology is one of the newest approaches with the potential to revolutionize the medical and pharmaceutical fields [19,20,74]. The incorporation of nanosystems in herbal medicine could serve as an effective tool in eradicating the limitations associated with medicinal plants [75]. With the emerging new technologies, these plants can be formulated into newer strategies that can enhance the delivery and efficacy of their phytochemicals [76]. Green nanotechnology has shown great promise in this regard and various medicinal plants have been reportedly used to synthesize MNPs with enhanced biocompatibility and biological activities [18,49]. Hembram et al. [77] reported the synthesis of AgNPs using aqueous leaf extracts of *Mentha pulegium*, *Coriandrum sativum*, and *Prosopis cineraria*. This particular study reported that the biogenic AgNPs showed an enhanced anticancer effect in in vitro breast cancer cells [77]. Due to the excellent pharmacological activities displayed by plant mediated MNPs, focus on how traditional plants would benefit the pharmaceutical industry especially in the drug discovery platform is now paramount.

South Africa is rich with a plethora of plants with a long history in traditional medicine for the treatment of infectious and chronic diseases, necessitating their inclusion in nanotechnology. The exploration of South African medicinal plants including *Camellia sinensis*, *Azadirachta indica*, *Aloe vera*, and *Jatropha curcas* plant extracts for the synthesis of a variety of MNPs has been documented. Moreover, a largescale screening of plant species obtained from the Cape floristic region of South Africa including *Aspalathus hispida*, *Indigofera brachystachya*, *Nidorella foetida*, and *Podocarpus falcatus* was successfully used for the synthesis of AuNPs [8]. Interestingly, the investigations regarding the activities of South African plant-mediated MNPs revealed enhanced antimicrobial and anticancer activities compared to the crude plant extract [6]. Despite the number of valuable indigenous plants utilized for MNP production, only very few of them have been explored for biological purposes. Therefore, the present study reviews 12potent plants commonly used in South Africa traditional medicine in terms of their potential for MNPs synthesis and their biological applications. Some of these plants are indigenous, while others are also found in other parts of the world. A list of some selected South African plants incorporating their traditional use for treating various ailments and their bioactivity through green nanotechnology is given in Table 1.

#### 4.2.1. *Cyclopia intermedia*

*C. intermedia*, commonly known as Honeybush (HB), is a popular South African shrub belonging to the family Fabaceae. The plant is an erect, much-branched shrub found in winter rainfall coastal and mountainous areas with a moderate Mediterranean climate [109]. It is a perennial plant with a woody stem that grows up to 1.5 to 3 m tall. HB is characterized by yellow flowers, long needle-like leaves with a honey-like smell [92,93]. The leaves and stems are used to brew the famous South African herbal HB tea known for several health benefits. Traditional healers from South Africa use the infusion prepared from the leaves and stem to relieve constipation, nervousness, cough, eczema, epilepsy, and to regulate blood pressure [21]. HB tea is caffeine-free and contains low tannin, making it a suitable tea that can be drunk at bedtime. The health benefit of HB include prevention of skin cancer and lowering of blood glucose levels [110]. Additionally, HB offers phytoestrogenic properties against breast cancer by binding to oestrogen receptor subtypes, thus, prevents the growth of breast cancer cells [111]. Its anticancer effects are through its modulation of oxidative stress, inhibition of cell proliferation, and adenosine triphosphate (ATP) production [111]. Studies showed that HB extracts contain polyphenolic compounds which are correlated with the plant’s biological activities. The significant compounds, characterized and isolated from HB, are the xanthones (MGF and isomangiferin) and flavanones (hesperidin, and isosakuranetin) [78,79]. These polyphenols offer the HB plant the ability to improve the immune system, protect against inflammatory diseases, and inhibit antimicrobial and tumour growth [110,111].

The synthesis of AuNPs using HB extracts (HBE) has been previously documented [20]. Using an eco-friendly approach, water extract of HB leaves was used to synthesize HB-AuNPs. The UV-Vis spectroscopic analysis showed that the biogenic HB-AuNPs displayed distinct peaks at 540 nm with a hydrodynamic diameter of 66.74 nm. The XRD analysis confirmed that the biogenic HB-AuNPs were crystalline. The average core size of the HB-AuNPs as determined by transmission electron microscope (TEM) was found to be 20 nm, exhibiting predominantly spherical with some triangular-shaped AuNPs. The FTIR analysis clearly showed the formation of AuNPs and indicated that HBE contains various phytochemicals, particularly polyphenols such as MGF, which could have been one of the phytochemicals that acted as reducing and stabilizing agents for the HB-AuNPs. A further study showed that the HB-AuNPs exhibited selective toxicity against brain (U87), prostate (PC-3), and colon (Caco-2) cancer cells [20]. These effects were compared and found similar to the effects of AuNPs synthesized from MGF (MGF-AuNPs). Thus, the study strongly suggests that MGF is possibly one of the reducing agents for the synthesis of HB-AuNPs [20]. Interestingly, the anticancer effects of both HB-AuNPs and MGF-AuNPs were further augmented when used in combination with doxorubicin [20]. Importantly, the study further investigated the effect of the HB-AuNPs and MGF-AuNPs on normal breast epithelial (MCF-12A) cells and showed that the biogenic MNPs had no toxicity on the cells even at the highest concentration of 1000 µg/mL [20]. This observation was consistent with earlier study that showed that 100 µM AuNPs synthesized using MGF (MGF-AuNPs) isolated from leaf extracts of M. indica L had no toxic effect on normal epithelial breast (MCF-10A) cells [51]. Similarly, Majoumouo et al. [19] showed that AuNPs synthesized using water extract of T. mantaly showed no significant reduction in cell viability of non-tumourigenic skin fibroblast (KMST-6) cells after 24 h treatment. Consequently, these findings present useful information in understanding the therapeutic application of biogenic MNPs, particularly in MNP formulations and dosage regimen.

#### 4.2.2. *Sutherlandiafructecens*

*S. frutescens* is an indigenous South African medicinal plant found predominantly in the Eastern, Northern, and Western Cape provinces, and some areas of KwaZulu-Natal. It is an attractive leguminous shrub that can grow from 0.5 m up to 1.2 m in height. Also, it has greyish-green leaves finely arranged in a feather-like feature. *S. frutescens* is characterized by transparent bladder-like fruits, large balloon-like seed pods, and orange-red flowers. Traditional healers in South Africa use decoction prepared from *S. frutescens* (leaves, stems, flowers, roots, and pods) to treat wounds, cancer, diabetes, rheumatism, influenza, gonorrhoea, and to reduce body temperature [6,80]. The plant is also used to alleviate diverse kinds of symptoms and conditions like depression and stress. Further, the plant is used to treat urinary tract infections, stomach infections, skin diseases, gonorrhea, kidney and liver problems [81]. The plant has been formulated into capsules, tablets, gels, ointments, and creams, and available in some pharmacies and herbal shops [112].

The phytochemical profile of the plant revealed the presence of saponins, pinitols, flavonoids, and triterpenoids, which are believed to be responsible for its enormous medicinal activities. Additionally, cannavanine, cycloartane glycosides, and flavonol glycosides are also abundantly present in the plant, contributing significantly to its bioactivity [80]. Pharmacological studies of *S. frutescens* extracts revealed evidence of its antiviral, anti-inflammatory, antibacterial, antiproliferative, antimutagenic, antioxidant, and antidiabetic properties [112,113]. An improved quality of life reported in HIV patients following treatment with commercial Sutherlandia tablets further expounded the popularity of the plant as an effective herbal product [113]. An earlier study reported a dose-dependent decrease in cell number and changes in the morphology of human breast cancer (MCF-7) cells following treatment with an aqueous extract of *S. fructescens* [113]. Moreover, the leaf extracts of *S. frutescens* reduced insulin levels and enhanced glucose uptake in streptotozin-induced diabetes in Wistar rats [114]. Owing to the acclaimed medicinal benefits, it is imperative to investigate the fabrication of *S. frutescens* extracts into MNPs for enhanced bioactivity.

**Figure 4:**
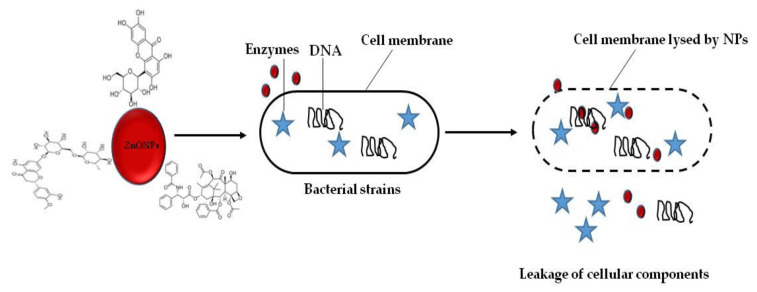
Mechanism of ZnO NPs against bacterial strains. ZnO NPs exhibited remarkable inhibition of cell growth and cell death in bacterial strains following the direct interaction between the ZnO NPs and the bacterial cell surface. Adapted from [81].

*S. fructescens* was first reported for the synthesis of Zinc Oxide (ZnO) NPs [81]. In this particular study, the water extracts of the plant were used to reduce zinc salt for the synthesis of ZnO NPs. The exact mechanism by which the ZnO NPs were formed is not yet fully understood. It was proposed that phytochemicals present in the plant donated hydrogen atoms, which resulted in the release of Zn^2+^ ion. Afterward, the Zn^2+^reacted with the polyphenols of the plant, resulting in the formation of ZnO NPs. The FTIR measurement revealed a vibrational peak at region 1400 cm^−1^ which corresponds to biomolecules that are involved in the formation of the ZnO NPs. The TEM images revealed spherical-shaped NPs with the core size ranging between 5 and 25 nm. The antibacterial investigation against *E. coli*, *S. aureus*, *P. aeruginosa*, and E. faecalis using agar well diffusion assay revealed that *S. frutescens* ZnO NPs inhibited the growth of all test strains. Although this particular study reported an impressive antibacterial activity for *S. frutescens* ZnO NPs, however, the mechanism of the antibacterial effect was not fully captured. Evidence shows that NPs can pass through the membrane to interact with cellular components of the bacterial cell wall, thus induce oxidative stress that subsequently leads to cell death (Figure 4). The *S. frutescens* ZnO NPs also exhibited a dose-dependent effect in lung (A549) cancer cells with approximately 93.4% cell death following a 24-h treatment [81]

*S. frutescens* leaf extracts were also used in the synthesis of AgNPs following incubation of water extract of S.frutescenswith AgNO_3_ in a simple and easy method [6]. The *S. frutescens* AgNPs were predominantly spherical and polygon-shaped and had an average core size between 15 and 20 nm. The various shapes and sizes indicated that more than one phytochemical was involved in the reduction and possibly capping of the AgNPs. This claim was validated by the overlapping peaks in the FTIR spectra of the extracts and the AgNPs. While the water extract *S. frutescens* showed antibacterial activity at concentrations up to 50 mg/mL, the biogenic AgNPs displayed a significant bacterial growth inhibition against *S. epidermidis* and *P. aeruginosa*. The minimum inhibitory concentration (MIC, lowest concentration that inhibits visible growth) of the AgNPs was ~66 fold lower than the concentration of the plant extract (50 mg/mL) that failed to inhibit growth [6].

#### 4.2.3. *Hypoxis hemerocallidea*

*H. hemerocallidea* of the hypoxidaceae family is an indigenous Southern African plant, found abundantly in South Africa, Swaziland, Lesotho, and Botswana. The plant, commonly known as ‘African potato’, ‘miracle plant’, ‘molic’, and ‘star flower’ is enlisted in Southern Africa as an indigenous medicinal plant with potential health benefits [83]. The plant is characterized by strap-like hairy leaves, yellow star-shaped flowers, and thick green hairy stems. Currently, *H. hemerocallidea* is commercialized as a natural product with potent applicability in drug development [23]. The traditional use of *H. hemerocallidea* as a strengthening tonic, purgative, and laxative, or to treat tuberculosis, urinary tract infection, infertility, cancer, diabetes, and wounds has been documented [82]. The promising anticancer properties reported for *H. hemerocallidea* prompted its inclusion as one of the South African plants used for the treatment of cancer in the Eastern Cape Province of South Africa. The popularity of the plant became more prominent following its recommendation for human immunodeficiency virus (HIV) patients as an immune booster [23].

The plant is composed of hypoxoside, which is believed to be the main component responsible for its numerous medicinal activities. Sterols, norlignane, daucosterols, stanols, hypoxide, sterolins, and β-sitosterol are also reported to play significant roles in the plants’ therapeutic activities. Also, *H. hemerocallidea* is laced with trace elements including, copper, zinc, and manganese, thus used as pro-fertility supplements. Evidence-based laboratory investigation indicated that extracts obtained from African potato possess numerous pharmacological properties including antidiabetic, anti-inflammatory, antioxidant, antihypeglycemic, analgesic, and anticancer [83].

The interesting biological properties entrusted in this plant led to its fabrication into MNPs with enhanced bioactivity. The aqueous leaf extract of *H. hemerocallidea* was explored in the synthesis of AuNPs (Hy-AuNPs). The reduction of the gold salt was achieved by the phytochemicals that contain the O-H, CH_3_, and C-O functional groups commonly found in flavonoids, terpenoids, carbohydrates, and phenolic compounds. The resulting AuNPs with hydrodynamic size range from 10–45 nm displayed selective effect against *P. aeruginosa*, *S. epidermidis*, *E. coli*, and *S. aereus* [27]. In another study, Elbagory et al. [27] compared the physicochemical properties of the Hy-AuNPs with AuNPs synthesized using water extract of hypoxoside (a compound isolated from *H. hemerocallidea*) and found that they were similar. This suggests that hypoxoside is involved in the synthesis of Hy-AuNPs. Furthermore, both AuNPs had immunomodulatary effects and reduced the pro-inflammatory cytokines in macrophages (THP 1) and natural killer (NK-92) cells [27].

A recent study by Aremu et al. [115] reported the successful synthesis of AgNPs using ethanolic extracts of *H. hemerocallidea*. The study showed that the resulting AgNPs with an average diameter of 6–20 nm exhibited significant antibacterial activity against *Bacillus cereus*, *Streptococcus pneumonia*, *P. aeruginosa*, *Moraxella catarrharis*, and *E. coli*. Interestingly, a combined effect of the AuNPs with a broad-spectrum antibiotic, streptomycin, showed that the AuNPs synergistically enhance the antibacterial effect of streptomycin up to 30–52% [115].

#### 4.2.4. *Eucomisautumnalis*

*E. autumnalis* of the Asparagaceae family is a deciduous, summer-growing bulb characterized by its extraordinary floral arrangement with wavy, soft, and fleshy flowers [86]. They are widely distributed in open grasslands and marshes across all the provinces of South Africa, as well as some neighbouring countries like Botswana, Zimbabwe, and Malawi [84]. Although the bulb is believed to be toxic if consumed on its own, a decoction prepared in water or milk is safe for treating fevers, urinary diseases, lower backaches, syphilis, and at times used to induce labour [84]. Many constituents, including homoisoflavanones, terpenoids, and diben-α-pyrones, have been reportedly isolated from this plant [85]. The traditional use of *E. autumnalis* as anti-inflammatory agents is popularly accepted, hence, it is recommended for the manufacture of non-steroidal anti-inflammatory drugs [84]. It is traditionally used to treat wounds, fractures and to relieve pain often associated with surgical procedures [84].

The phytochemicals present in water extract of *E. autumnalis* plant were used for the reduction of AgNO_3_ to AgNPs [86]. The resulting brownish-black colour connotes the formation of AgNPs [40]. The UV-Vis spectrophotometric analysis of the formed MNPs gives SPR bands at 423 nm, confirming the presence of AgNPs.FTIR analysis of biosynthesized AgNPs signals the contribution of olyphenols and aromatic compounds in the bioreduction process. The biosynthesized *E. autumnalis* AgNPs revealed the hydrodynamic size of 56 nm and predominantly spherical shape AgNPs. These AgNPs were further evaluated for their antimicrobial activities against Listeria monocytogenes, Enterococcus faecalis, K. pneumonia, and Acinebacter baumannii. The result indicates that the AgNPs displayed enhance antimicrobial effects over its bulk plant extracts in all test stains [86]. Consequently, this finding revealed that *E. autumnalis* AgNPs have promising antibacterial properties against bacteria strains.

#### 4.2.5. *Plumbago auriculata*

*P. auriculata* is a bushy evergreen shrub belonging to the family Plumbaginaeae. It is a native South African plant but is also distributed in other tropical and subtropical regions of the world, including Sri Lanka and India [88]. In South Africa, *P. auriculata* is predominantly found in the Southern and Western Cape to the balmy subtropical province of Kwazulu-Natal. It is commonly known as Cape leadwort in English or blousyselbos in Afrikaans [87]. Traditionally, *P. auriculata* is used to treat headaches, warts, skin infections, wounds, and fractures [88]. The plant is reported to contain a wide range of phytochemicals, including tannins, phenols, alkaloids, saponins, flavonoids and, proteins. Bioactive compounds including plumbagin, α-amyrin, capensisone, and diomuscinone have been isolated from the plant [88]. A study shows that most of the pharmacological activity, including antimicrobial, anticancer, and anti-inflammatory effects of the plants, is conferred by its biomarker compound plumbagin [88]. Plumbagin is an effective inhibitor of cell growth, displaying a broad-spectrum anticancer effect against a wide range of cancers, including breast, liver, prostate, pancreatic, and ovarian cancers [88].

Earlier, the successful synthesis of AgNPs from *P. auriculata* leaf and calyx extracts had been reported [7]. In the study, the bioreduction of Ag+ ions by the aqueous extracts was demonstrated in an eco-friendly manner. In the process of synthesis, a colour change from yellowish-brown to dark brown and light yellow to dark brown was observed for the leaf and calyx extracts, respectively. The colour change confirmed the bioreduction of Ag^+^ to Ag^0^ [35]. The biogenic AgNPs so produced denoted by the appearance of absorption peaks between 420 and 460 nm. The AgNPs were relatively spherical to oblong in shape with a core size diameter of 15.22 and 26.5 nm for leaf and calyx extracts, respectively. FTIR spectra revealed that the phytochemicals containing -OH and C=O groups in both extracts were involved in the bioreduction and stabilization of AgNPs [35]. The antibacterial activities of both biogenic AgNPs revealed their desirable antibacterial effects against *E. coli*, *K. pneumoniae*, *S. typhimurium*, and *S. aureus* as compared to their respective bulk plant extracts. The study was the first to report the antibacterial activities of AuNPs using water extracts of *P. auriculata* against gram-positive and gram-negative multidrug resistance bacteria [7]. Consequently, the effective antibacterial activity of the biogenic AuNPs opens a new path in antibacterial drug discovery.

#### 4.2.6. *Catharanthus roseus*

*C. roseus*, also known as Madagascar periwinkle or Vinca rosea, is a well-studied medicinal plant. It is a perennial herb characterized by a woody base with glossy bright green leaves and pink to white flowers [116,117]. *C. roseus* originated from Madagascar and was imported to South Africa as an ornament, but has now become widely distributed in major parts of South Africa including KwaZulu-Natal, Limpopo, and Gauteng Provinces [118]. *C. roseus* has been extensively studied and shown to contain over 130 phytochemicals with vindoline implicated as its principal marker compound [119]. Other major constituents isolated from *C. roseus* include vincristine and vinblastine which are well-known anticancer drugs used for treating Hodgkin’s lymphoma and leukemia [120]. Most of the alkaloids isolated from this plant include deoxyvinblastine, vincoline, cathanranthamine, rosicine, and leurosine have diverse pharmacological activities [117].

Traditionally, a decoction made from leaf extracts is used to treat rheumatism, venereal diseases, skin infections, high blood pressure, diabetes mellitus, and many others [26,90]. In Madagascar, the extract from the plant is used as purgative, vomitive, and for relieving toothache [118,121]. Antioxidant and pharmacological activities of C. rosues such as antidiabetics, antimicrobial and anti-inflammatory activities, have been reported. The wound healing potential of ethanolic leaf extracts of *C. roseus* showed an enhanced wound contraction in Wistar rats [122]. Verma and Singh [121] reported the antimicrobial influence of leaf extract of *C. roseus* against *S. citreus*, *S. aureus*, *E. coli*, and *P. aeruginosa*, indicating *C. roseus* as an excellent antibacterial agent.

Different parts of *C. roseus*, including stem, root, leaf, and flower, have been explored for the synthesis of AgNPs. For example, an eco-friendly synthesis of AgNPs using aqueous leaf extract of *C. roseus* was reported [41]. Here, the bioactive constituents of *C. roseus* leaf extracts were used to reduce silver salt for the synthesis of *C. roseus* AgNPs. The observed colour change from light yellow to brown following incubation of *C. roseus* leaf extract and AgNO_3_ solution indicated the reduction of Ag+ to AgNPs. This was confirmed by the appearance of an absorption peak at 425 nm. Additional characterization revealed biosynthesized AgNPs of an average size of 49 nm having crystalline nature. FTIR techniques adopted to investigate the plausible mechanism behind the formation of these AgNPs revealed the presence of amide group possibly contributed by enzymes/proteins present in the plant extract. These compounds were proposed to be responsible for the reduction of AgNO_3_ to AgNPs. The antioxidant activity of the biogenic crystalline AgNPs investigated using 2,2-Diphenyl-1-picrylhydrazyl (DPPH)-free radical scavenging assay showed a dose-dependent increase in the DPPH inhibition percentage up to 82% at 300 µg/mL. Also, these AgNPs showed good antimicrobial effects against *E. coli*, *K. pneumoniae*, *P. aeriginosa*, *S. aureus*, *C. koseri*, and *C. albicans* when compared to the plant extract or AgNO_3_ alone [75]. Further study revealed the wound-healing activity of the biosynthesized NPs as it had enhanced wound closure and reduced wound size in male albino mice compared to untreated animals with no evidence of microbial contamination [41].

Another study reported an eco-friendly approach for the synthesis of AgNPs from the aqueous root extract of *C. roseus* in the presence of silver nitrate [26]. A colour change from light yellow to dark brown indicated the presence of AgNPs, which was confirmed by SPR at 423 nm. The resulting spherical shaped AgNPs with sizes ranging between 35 and 55 nm. FTIR analysis indicates the presence of alkanes and aliphatic amines, which were believed to be responsible for the bioreduction and stabilization of the AgNPs. These AgNPs were evaluated for their larvicidal activity against fourth instar larvae of Aedes aegypti and Culex quinquefasciatus and it was observed that the biogenic AgNPs had enhanced larval mortality effect when compared to *C. roseus* root extracts [26]. Thus, the study revealed the potential of AgNPs synthesized using *C. roseus* as strong anti-larvalcidal agents.

Similarly, AgNPs synthesized using an aqueous extract of *C. roseus* flower were reported. FTIR spectra indicate the presence of aldehydes, carbonyls, alcohols, phenols, alkanes, and aliphatic amines. The spherical-shaped biosynthesized AgNPs having an average size ranging between 6 and 25 nm showed remarkable antibacterial activity against *E. coli*, *S. aureus*, *K. pneumonia*, and *B. subtilus* [75]. Other parts of *C. roseus* including stem have also been explored for the synthesis of AgNPs and their antimicrobial activity has been documented. Given the high efficacy of AgNPs of *C. roseus* as strong antibacterial, antioxidant, antilaraicidal, and wound healing agents, it is recommended that other biological applications including anticancer property and the synthesis of other metallic NPs should be investigated.

#### 4.2.7. *Aspalathus linearis*

*A. linearis*, belonging to the family Fabaceae, is an endemic South African plant widely distributed in the Western Cape Province of the country. *A. linearis* is cultivated to brew the popular herbal Rooibos tea. It is frequently used traditionally to treat insomnia, stomach cramps, allergies, digestive problems as well as to improve appetite [21]. Previous and recent in vitro and in vivo studies have implicated *A. linearis* as a rich source of phenolic compounds. Bioactive compounds viz, aspalathin, orientin, isoquercitrin, luteolin, and hyperoside have been reportedly isolated from *A. linearis* [78,93,111]. These are believed to be responsible for their antioxidant, immunomodulatory, anti-inflammatory, antidiabetic, and chemoprevention effects [93].

The synthesis of AuNPs using *A. linearis* tea leaves to optimize the antifungal activity of some commercial antifungal discs has been documented [87]. In this study, a green route approach was attempted for the synthesis of AuNPs from the *A. linearis* plant where the aqueous plant extract was mixed with NaAuCl_4_ and stirred for 30 min at room temperature. A colour change from yellow to ruby-red in the reaction mixture indicating the formation of AuNPs showed a strong resonance peak at 529 nm. The biogenic AuNPs with an average size of 44 nm, demonstrated excellent in vitro stability in different biological media at varying pH levels [94]. The resulting biogenic AuNPswere investigated for their enhanced antifungal activities against Aspergillus spp using disc diffusion assay. In this particular study, the AuNPs were coated around eight commercial antifungal discs (clotimazole, nystatin, flucytosine, fluconazole, econazole, ketoconazole, miconazole, and amphotericin) and their zones of inhibition were compared to those coated with pristine. The AuNPs attached better onto the antifungal disc compared to the pristine coated antifungal disc resulting in an enhanced antifungal activity against Aspergillus spp. This study revealed AuNPs as good antifungal agents that could be of importance in medical and veterinary applications. The later part of their study investigated the toxicity of the biosynthesized AuNPs against human Hep-G2 liver cancer cells, and the result indicated that the AuNPs showed no significant effect on the cells [94].

The aqueous leaf extract of *A. linearis* was also used for the synthesis of rhodium (Rh) NPs at room temperature [29]. The disappearance of the deep orange colour typical of Rh precursor to a lit orange, following the addition of the Rh precursor to the aqueous extract of *A. linearis*, indicated the formation of RhNPs [29]. The *A. linearis* extracts showed near-infrared (NIR) absorbance in the optical range of 225–600 nm, however, the peak almost completely disappeared in the presence of the RhNPs which is as a result of the reduction of RH^3+^ to Rh^0^ [29]. The RhNPs were non-agglomerated and had an average size of approximately 1.2 nm. Further characterization revealed that the RhNPs were amorphous [29]. Apart from RhNPs, other metallic NPs, including zinc oxide and cobalt, have been reportedly synthesized from *A. linearis* and fully characterized [123], but nothing is reported regarding their biological applications.

#### 4.2.8. *Indigofera tinctoria*

*I. tinctoria*, belonging to the Fabaceae family, is an erect, branched perennial plant found predominantly in widespread tropical Africa. The plant is characterized by its greyish-brown stem and dark-green leaves [124]. *I. tinctoria* species is reported to be a major source of the colour indigo, and the leaves have widely been cultivated, extracted, and processed for the production of indigo dye [95]. Leaf infusion of *I. tinctoria* is traditionally used to treat epilepsy, asthma, stomachache, bronchitis, and some skin diseases. An investigation of its phytochemical contents revealed that the plant contains reducing sugars, saponins, carbohydrates, alkaloids, flavonoids, and phenolic compounds [96].

Recently, Vijayan et al. [97] reported the first-time synthesis of AuNPs from aqueous leaf extract of *I. tinctoria*. A change in colour from light yellow to violet after 30 s of exposure to microwave irradiation was observed for the AuNPs, which was later confirmed by an SPR peak at 545 nm. Further characterization regarding the morphology of the AuNPs revealed that the NPs were crystalline in nature, triangular, spherical, and hexagonal, and having a particle core size ranging between 6 and 29 nm [97]. An investigation of the antibacterial and antifungal activities of the AuNPs revealed some degree of antimicrobial effects against *B. pumilis*, *S. aureus*, *P. aeruginosa*, *E. coli*, *Aspergillus fumigatus*, and *Aspergillus niger* with noticeable zones of inhibition. In addition, the study further evaluated the anticancer effect of *I. tinctoria* AuNPs on human lung (A549) cells using the 3-[4,5-dimethythiazol-2yl]-2,5 diphenyl tetrazolium bromide (MTT) assay. The result revealed that the biogenic *I. tinctoria* AuNPs showed enhance toxicity against lung cancer (A549) cells with evident changes (shrinking) in cellular morphology compared to the bulk plant extract [97]. Therefore, the findings from this study clearly showed that AuNPs synthesized from water extracts of *I. tinctoria* can be effectively used as powerful weapons against cancer cells, particularly lung cancer as well as against bacteria cells.

#### 4.2.9. *Artemisia herba-alba*

*A. herba-alba*, also known as the white wormwood, is a perennial shrub that grows commonly on the dry steppes of the Mediterranean regions in Northern Africa, Western Asia, and South-Western Europe [21]. Its leaves are strongly aromatic and covered with fine glandular hairs. It is used as an antiseptic and antispasmodic, as well as a treatment for anorexia, indigestion, and gastrointestinal problems [99]. Major constituents of *A. herba-alba* include 1,8-cineole and appreciable amounts of alpha and beta-thujone as well as other oxygenated monoterpenes in addition to davanone, chrysanthenone, and cis-chrysanthenol [125]. Phytochemical investigation of the aerial part of *A. herba-alba* revealed two new natural sesquiterpene lactones [102] in addition to artemisin, an endoperoxide sesquiterpene lactone and the quaianolide structural type. These compounds, particularly those isolated from its essential oil, tend to provide Artemisia with a wide range of bioactivity, including antibacterial, antiseptic, antifungal, and choleretic activities [126]. *A. herba-alba* is commonly used as a remedy for enteritis, menstrual pain, nervous problems, and various intestinal disturbances among the Bedouins in the Negev desert of Israel [21,101]. Essential oil from this plant showed antibacterial and antispasmodic activities in rabbits [120]. Also, an aqueous extract of aerial parts of the plant with a hypoglycemic effect in alloxan-induced diabetic rabbits and mice has been reported [98].

The first record of *A. herba-alba* mediated synthesis of AgNPs was reported [101]. The appearance of a yellowish-brown suspension following the mixing of AgNO_3_ solution with the water extracts of *A. herba-alba*, in addition to the peak at 425 nm, indicated the formation of AgNPs. Further characterization of the fabricated *A. herba-alba* AgNPs revealed spherical shaped NPs with a size range of 43 and 74 nm. FTIR analysis of the fabricated AgNPs indicated relevant absorption peaks corresponding to N-H stretching from peptide linkages and C-C stretching vibrations of aromatic amines. The fabricated AgNPs were tested on Indian and Saudi Arabian strains of Anopheles, Aedes, and Culex mosquitoes and reported to be a source of green nanoinsecticides against mosquito vectors [123]. Also, the bacteria growth inhibition effect of the biogenic *A. herba-alba* AgNPs was demonstrated against *B. subtilis*, *K. pneumonia*, and *S. typhi*. This study proposed that the fabricated *A. herba-alba* AgNPs have relevant insecticidal and bactericidal activities against species of high public health importance [101].

#### 4.2.10. *Cantella asiatica*

*C. asiatica* is a herbaceous, perennial plant native to South Africa, Asia, and the South Pacific. In South Africa, the decoction of *C. asiatica* is used to treat fever, leprosy, syphilis, tuberculosis, asthma, epilepsy, mental disorder, minor wounds, and it is also consumed as a vegetable and used as a spice [21]. *C. asiatica* has been extensively studied to contain pentacyclic triterpenoids, centellose, centelloside, and medacassoside, which are believed to proffer its healing potential [103]. The major chemical constituents found in this plant are triterpene saponosides, flavonoid derivatives viz quercetin, rutin, kaemferol, patuletin, apigenin in addition to polysaccharides, polyacetylenes, phenolic acids, and sterols [103]. An investigation of the neuroprotective activity of *C. asiatica* demonstrated that the plant can inhibit acetylcholinesterase, a key enzyme implicated in the pathogenesis of Alzheimers disease [127]. Moreover, the wound healing property of ointment, cream, and gel formulated from aqueous extract of this plant showed enhanced wound healing properties in Wistar rats by increasing collagen content and tensile strength at the wound site [128]. Other studies also documented the cognitive-enhancing, antioxidant, antidepressant, antiepileptic, sedative, and anxiolytic properties of *C. asiatica*.

The synthesis of AgNPs using aqueous leaf extracts of *C. asiatica* L. was developed by Rout and colleagues [104]. Upon incubation of plant extracts with silver salt at room temperature, stable AgNPs were produced without the involvement of toxic chemicals as capping agents. Biosynthesized AgNPs were spherical with a size range between 30 and 50 nm. Agar well diffusion assay revealed effective antimicrobial activity of biosynthesized *C. asiatica* AgNPs against *S. aureus* [104].

In a similar study, Thakkar et al. [62] synthesized AgNPs using aqueous leaf extracts of C. asiatica. In this particular study, the antimicrobial activity of both the aqueous extracts of *C. asiatica* and its resulting AgNPs were compared against *S. aureus*, *P. aeruginosa*, and *E. coli*. Results showed that leaf extracts of *C. asiatica* had low activity compared to those exhibited by its biogenic AgNPs. This finding suggests that *C. asiatica* AgNPs interact with the bacteria cell wall more than the extracts and thus describe the AuNPs as stronger antimicrobial agents compared to its bilk plant extract [62].

#### 4.2.11. *Galenia africana*

*G. africana* is a major group of flowering plants belonging to the family Aizoaceae. *G. africana* commonly referred to as kraalbos, geelbos, or perdebos, is found abundantly in Namaqualand, Northern Cape, some parts of Karoo, and Eastern Cape Province of South Africa. The plant is characterized by leaves growing in the opposite direction, soft woody shrublet (0.5–1.5 m high), and acute apex flowers. The plant is used to treat venereal sores, eye infections, asthma, tuberculosis, cough, wounds, and skin infections. Locals masticate the leaves of *G. africana* to relieve toothache [105]. The earlier phytochemical investigation reported by Mativandlela et al. showed three known flavonoids viz, trihydroxyflavanone, trihydroxychalcone, dihydroxychalcone, and a new trihydroxy-3-methoxychalconeare present in the *G. africana* [106].

The first-time synthesis of AuNPs from *G. africana* was demonstrated by [25], where the phytochemicals present in aerial parts of *G. africana* were utilized for the reduction of gold salts to form Galenia-AuNPs. The change in colour from light yellow to red colour following a 1 h incubation at 70 °C indicated the appearance of Galenia-AuNPs [25]. The AuNPs had absorption spectra at 534 nm with varying shaped crystalline AuNPs with core sizes ranging between 9 and 27 nm. The different shapes were an indication that different bioactive compounds present in the plant extract actively participated in the reduction process as equally revealed by FTIR. The biogenic AuNPs were stable in various biological media and exhibited antibacterial effects against *S. aureus*, *E.coli*, *S. epidermidis*, and *P. aeruginosa*. These microbes are very common in wound infections, consequently Galenia-AuNPs could be recommended as potent treatment regimen for wound infections. To address the toxic effects of biogenic AuNPs in non-cancer cells, this particular study further reported that the Galenia-AuNPs have excellent biocompatibility in KMST-6 cells [25]. The Galenia-AuNPs at a concentration of 32 µM following a 24 h treatment showed no significant reduction in the cell viability [25]. On the other hand, Hossain et al. [129] investigated the toxic effect of biogenic AgNPs synthesized from water extract of Andrographis paniculata stem in healthy male Wistar rats. The result showed that there was no significant toxic effect on the liver and kidneys following an intravenous treatment of the rats with either 2 mg/kg or 5 mg/kg body weight. More specifically, there was no significant difference in serum biomarkers and creatine levels between the treated and control animals [129]. Thus, biogenic MNPs are believed to offer excellent biocompatibility and can be recommended for future therapeutic applications [25].

#### 4.2.12. *Sclerocarya birrea*

*S. birrea* is a medium-sized to a large deciduous tree with an erect trunk, having its leaves mostly crowded at the end of the branches. The plant is commonly found in riverine, grasslands, and bushlands as well as in well-drained, loamy, and sandy soil [67]. *S. birrea* is predominantly found in the Phalaborwa area in Limpopo [107]. Traditionally, the decoction of *S. birrea* is used to treat dysentery, rheumatism, malaria, and diarrhea [108]. The leaves are eaten as vegetables, while the fruits and plums, including alcoholic drinks, jams, and juice produced from the plant, are consumed in most countries of West Africa [108]. Medicinal value credited to this plant is traced to their secondary metabolite contents, including glucosides, steroids, glycosides, flavonoids, fatty oils, alkaloids, phenols, resins, calcium, and phosphorus. Moreover, traditional healers in West Africa use the infusion prepared from the stem bark of the plant to treat diabetes [108].

In an earlier study, Lediga and colleagues explored the phytochemicals present in *S. birrea* plant extracts for the synthesis of AgNPs [86]. The resulting brownish-black colour in the reaction medium confirmed the formation of AgNPs which was confirmed by the SPR band at 440 nm. The FTIR analysis of biosynthesized AgNPs indicated the presence of polyphenols and aromatic compounds, which were believed to play major roles in the bioreduction process. The hydrodynamic size of 112 nm for *S. birrea* AgNPs with predominantly spherical NPs. The AgNPs NPs were evaluated for their antimicrobial activities against *L. monocytogenes*, *E. faecalis*, *K. pneumonia*, and *A. baumannii*. The result displayed the enhanced antimicrobial effect of *S. birrea* AgNPs over its bulk plant extracts exhibiting the highest inhibition against A. baumannii [86].

### 4.3. Preclinical and Clinical Application of MNPs

Despite the highlighted potential of biosynthesized MNPs, very few of them have been critically evaluated for in vivo purposes [130] and only one was evaluated in human trials [73]. This suggests that many of the MNP formulations have not sufficiently accomplished the pharmaceutical regulation to warrant their clinical application [131]. Nonetheless, pre-clinical studies so far have provided compelling evidence of the potential clinical application of the biogenic MNPs. Some of their biomedical applications are highlighted in Section 2, where they are used as antimicrobial, wound healing [41], drug delivery [20,73], and therapeutic agents [19,28]. The outcomes of these nanomaterials, supported by the longstanding use of the medicinal plants further encourage their translation into clinical practice. In fact, the biogenic AgNPs have potential to replace the chemically synthesized MNPs due to their biocompatibility. Additionally, the biosynthesized MNPs are capable of targeting and killing diseased cells without attaching targeting and therapeutic moieties [19].

The wound healing property of biogenic *C. roseus* AgNPs was reported in mice. Further, the study observed sign of microbial contamination, pus formation, inflammation and bleeding in untreated mice, whereas, mice treated with the *C. roseus* AuNPs showed none of these symptoms [41]. Independent studies demonstrated the anti-tumor effect of AuNPs loaded with resveratrol (RES-AuNPs) in breast (MDAMB-231), pancreatic (PANC-1), prostate (PC-3) cancer cells [76], as well as in mice bearing liver tumours [132]. In both studies the RES-AuNPsexhibited stronger anti-tumour effect when compared to free resveratrol [76,132]. MGF-AuNPs were the extensively studied, and demonstrated to have antitumor effects on various cancer cells alone [74] or in combinanation with chemotherapeutic drugs [20]. MGF-AuNPs was also shown to significantly reduce tumour volumes in mice implanted with PC-3 tumour xenografts within three weeks of administration. This particular study further revealed that 80% of the intratumoural administered radiolabelled MGF-AuNPs were localized in the tumour site within 30 min of treatment and this was maintained for 24 h [74]. These encouraging pre-clinical results prompted the work of Khoobchandani et al. [73] that investigated the clinical efficacy of biogenic MNP formulation in metastatic breast cancer patients. The study established Nano Swarna Bhasma (NSB), a nanodrug prepared through the proprietary combinations of phytochemicals from Emblica officinalis, Mangiferaindica, Curcumin longa, Acacia nilotica, and Glycyrrhiza glabra with the AuNPs synthesized using Mangifera indica peel extract [73]. The NSB exhibited selective toxicity against human breast (MDA-MB-23) cancer and non-cancer aortic endothelial (HAEC) cells [73], and reduced tumour size in Severe Combined ImmunoDeficient mice. More importantly, the study also reported significant therapeutic benefits in breast cancer patients treated with NSB and these benefits were more pronounced when used in combination with antitumour drugs (doxorubicin and cyclophosphamide). Although, NSB is the first biogenic MNP to be used in human trials [73], there are a number of other MNPs that have been approved by the Food and Drug Administration (FDA) for clinical trials. These include AuNPs approved for cancer (NCT00356980), diabetes (NCT02837094), dental (NCT03669224), and skin (NCT02219074) therapy; AgNPs for skin infections (NCT03752424); and iron oxide NPs for cancer therapy (NCT02033447) and imaging (NCT04261777).

## 5. Conclusions and Future Perspectives

It is evident from literature that biogenic MNP formulations undeniably have broad-spectrum propensity against a wide array of diseases, however, their translation into conventional medicine suffers from a number of drawbacks, particularly the lack of information regarding their fate in vivo. According to literature, various biological models incorporating plants, mammalian cells, microorganisms, fish and mammals have been used to evaluate the toxicity of plant mediated MNPs, yet none has been able to sufficiently establish the exact mechanism involved in their toxicity. On the other hand, quite a number of pre-clinical/clinical investigations have evidently reported interesting antimicrobial, wound healing, anticancer, antioxidative, anti-inflammatory, immunomodulatory, and cytotoxic activities of these biogenic MNPS. It is therefore recommended that considerable efforts need to be devoted towards assessing the efficacy and safety of these natural MNP formulations in clinical research. More importantly, detailed description of the methods for the synthesis, purification, exact composition of the MNP formulations as well as the dosage should strictly be provided to allow for its reproductivity. In this regard, MNP formulations composed mainly of biological material could serve as better options over chemically synthesized MNPs, thus their translation into conventional medicine could be much faster, with the benefit to overcome the many challenges associated with the present-day standard modes of treatment.

## Figures and Tables

**Table 1:** List of selected South African medicinal plants utilized for MNP synthesis and their biological activity.

Plant Species	Indigenous Application	Major Phytochemicals	MNPs	MNP Size (nm)	MNP Bio-Activity
*Cyclopia intermedia*	Treat constipation, nervousness, cough, eczema, epilepsy and regulate blood pressure [21]	Mangiferin (MGF), isomangiferin, hesperidin and isosakuranetin [78,79]	AuNPs	20	Anticancer [20]
*Sutherlandiafructecens*	Treat wounds, cancer, diabetes, skin diseases, rheumatism, urinary tract infection, fever, gonorrhoea, kidney and liver problems [6,80]	Saponins, pinitols, flavonoids, triterpenoids, Cannavanine, cycloartane glycosides, flavonol glycosides, and aminobutyric acid [80]	ZnONPs AgNPs	5–25 15–20	Antimicrobial [81] Antibacterial and anticancer [6]
*Hypoxishemerocallidea*	Immune booster, purgative, and laxative tonic Treat tuberculosis, urinary tract infection, infertility, cancer, diabetes, and wounds [82,83]	Sterols, norlignane, daucosterols, stanols, hypoxide, sterolins, and β-sitosterol [82]	AuNPs	9–27	Antibacterialand anti-inflammatory [25,27]
*Eucomisautumnalis*	Reduce fever, urinary diseases, stomach, lower backaches, syphilis and sometimes used to induce labour [84,85]	Homoisoflavanones, terpenoids, and diben-α-pyrones [85]	AgNPs	56	Antimicrobial [86]
*Plumbago auriculata*	Treat headaches, warts, skin infection, wounds, and fracture [87,88]	Tannins, phenols, alkaloids, saponins, flavonoids, plumbagin, α-amyrin, capensisone, and diomuscinone [87]	AgNPs	15.22	Antimicrobial [7]
*Catharanthus roseus*	Treat rheumatism, venereal diseases, skin infections, high blood pressure, and diabetes [89,90]	Vinblastine, deoxyvinblastin, vincoline, cathanranthamine, rosicine, leurosine, vindoline, vincristine [91]	AgNPs AgNPs AgNPs	49 35.55 6–25	Antimicrobial and wound healing [41] Larvicidal [26] Antimicrobial [75]
*Aspalathus linearis*	Treat insomnia, stomach cramps, allergies, digestive problems as well as improve appetite [22]	Spalathin, orientin, isoquercitrin, luteolin hyperoside [92,93]	AuNPs RhNPs	44 1.2	Antimicrobial [94]
*Indigofera tinctoria*	Epilepsy, asthma, stomach ache, bronchitis, and some skin diseases [95]	Saponins, alkaloids, flavonoids, phenolic compounds [96]	AuNPs	6–29	Antibacterial, antifungal, and anticancer [97]
*Artemisia herba-alba*	Treat anorexia, indigestion, and gastrointestinal problems [98,99]	1,8-cineole, alpha, and beta-thujone, davanone, chrysanthenone, cis-chrysanthenol [98,100,101]	AgNPs	6–29	Antibacterial and mosquito repellant [102]
*Centella asiatica*	Treat fever, leprosy, syphilis, tuberculosis, leprosy, asthma, epilepsy, mental disorder, minor wounds Consumed as a vegetable and used as a spice [103]	Triterpenoids, centellose, medacassoside, triaponosides, flavonoid quercetin, rutin, kaemferol, patuletin, apigenin, polyacetylenes, phenolic acids, sterols [103]	AgNPs	30–50	Antimicrobial [104]
*Galenia africana*	Treat venereal sores, eye infections, asthma, tuberculosis, cough, wounds, skin infections and relieve toothache [105]	Trihydroxyflavanone, trihydroxychalcone, dihydroxychalcone, trihydroxy-3-methoxychalcone [106]	AuNPs	9–27	Antibacterial [25]
*Sclerocarya birrea*	Treat dysentery, rheumatism, malaria, and diarrhea [107,108]	Glucosides, steroids, glycosides, flavonoids, fatty oils, alkaloids, phenols, resins, calcium, phosphorus [107,108]	AgNPs	112	Antimicrobial [86]

## Data Availability

Not applicable.
